# Multivariate models of inter-subject anatomical variability

**DOI:** 10.1016/j.neuroimage.2010.03.059

**Published:** 2011-05-15

**Authors:** John Ashburner, Stefan Klöppel

**Affiliations:** a Wellcome Trust Centre for Neuroimaging, 12 Queen Square, London, WC1N 3BG, UK; b Department of Psychiatry and Psychotherapy, Section of Gerontopsychiatry and Neuropsychology, Freiburg Brain Imaging, University Hospital Freiburg, Freiburg, Germany

## Abstract

This paper presents a very selective review of some of the approaches for multivariate modelling of inter-subject variability among brain images. It focusses on applying probabilistic kernel-based pattern recognition approaches to pre-processed anatomical MRI, with the aim of most accurately modelling the difference between populations of subjects. Some of the principles underlying the pattern recognition approaches of Gaussian process classification and regression are briefly described, although the reader is advised to look elsewhere for full implementational details. Kernel pattern recognition methods require matrices that encode the degree of similarity between the images of each pair of subjects. This review focusses on similarity measures derived from the relative shapes of the subjects' brains. Pre-processing is viewed as generative modelling of anatomical variability, and there is a special emphasis on the diffeomorphic image registration framework, which provides a very parsimonious representation of relative shapes. Although the review is largely methodological, excessive mathematical notation is avoided as far as possible, as the paper attempts to convey a more intuitive understanding of various concepts. The paper should be of interest to readers wishing to apply pattern recognition methods to MRI data, with the aim of clinical diagnosis or biomarker development. It also tries to explain that the best models are those that most accurately predict, so similar approaches should also be relevant to basic science. Knowledge of some basic linear algebra and probability theory should make the review easier to follow, although it may still have something to offer to those readers whose mathematics may be more limited.

## Introduction

In recent years, the neuroimaging field has begun to see an increasing popularity in the use of modelling approaches that are multivariate over space. Rather than testing hypotheses about regionally specific effects using mass-univariate statistical models, such techniques attempt to combine all the data into the same model. Such an approach may be able to uncover unpredicted patterns that could otherwise be overlooked. The field of multivariate modelling is extremely large, so the current manuscript will be limited to a small subset of approaches for classification and regression. One particular strategy for modelling inter-subject anatomical variability will also be emphasised. Practical real world applications of pattern recognition models are obvious, but their contribution to our understanding of neuroanatomical variability may be less clear, so a small section on visualising differences is included.

Scientific research is usually dichotomised into the domains of *basic* (also known as *fundamental* or *pure*) and *applied* research. More recently, the concept of *translational* research has arisen within biomedical sciences, which is an alternative paradigm based upon a more seamless integration of the two traditionally separate domains of basic and applied research. Many consider basic research as simply “not-yet-applied”, which broadly agrees with the mission statements of the bodies that fund neuroimaging research.

When science is applied, it generally involves the use of models to make predictions, where these predictions may inform some decision making process. The ability to predict the behaviour of a system should enable interventions to be made that are more likely to cause favourable outcomes, where the favourability may be defined explicitly according to some utility function. For example, in a medical situation the objective would be to treat the patient to optimise life expectancy and quality of life measures, as well as financial and other considerations. Clinical intuition often conflicts with the optimal approach to decision making (Elstein and Schwarz, 2002), although evidence based medicine prescribes the use of Bayes theorem in order to overcome the various cognitive biases. The ability to predict is also useful for other translational areas such as pharmaceutical development, where decisions need to be made, such as those about which candidate drugs are most likely to succeed. One of the areas where brain imaging appears to offer the greatest potential contribution to translation, is in the area of imaging biomarkers.^1^ A useful imaging biomarker would have the ability to predict the eventual outcome of treatment, before the final end point criterion is reached.

Biology is not an exact science, so ideally such predictions should be probabilistic in order to encode the distribution of possible results. By predicting the probability over which events may occur, a model is also saying which events ought not to occur, or are less probable. Model predictions are therefore needed to ensure that claims are falsifiable. This paper will present a Bayesian perspective for validating claims, which involves comparing alternative models and selecting the one with the greatest evidence.

This journal is largely about basic neuroscience research, where the aim is to model the brain at the systems level. Having an accurate model of the system allows perturbations to be made to the model so the effects of similar perturbations may be anticipated in the real world. The usual aim of systems biology is to take a holistic view of modelling, which attempts to integrate data from a diverse range of sources. The various “omics” techniques, form a key component of systems biology — along with the associated informatics and data mining procedures required for identifying hidden patterns in the data (Kitano, 2002). Systems biology essentially involves an attempt to reverse engineer the system under study, where the end result is an accurate and useful model (Markram, 2006). Typically, it takes several decades for basic research to become applied, and the development of fully integrated models of the brain is still in the very tentative stages (Mazziotta et al., 2001; Oishi et al., 2008; Smith et al., 2009; Stephan et al., 2001). Every claim made by a scientist is in the context of some model or other, so findings pertaining to differences among populations of subjects need to be interpreted within the context of a model of inter-subject variability.

Many models can be thought of as *generative models*, as they allow samples to be generated from the probability density they encode. Such samples may be considered as realisations of the data, as simulated by a Bayesian model. There are plenty of arguments in favour of adopting a Bayesian view of modelling. Through the use of Dutch Book arguments, Bruno de Finetti showed that the Bayesian probabilistic framework provides the optimally coherent system for predictive inference (see e.g. Jaynes and Bretthorst (2003)). Within de Finetti's framework, Bayesian models can be conceptualised as a way of encoding probabilistic predictions about future observations, such that probabilities are represented over a whole range of possible outcomes. Probability densities learned by the models may be made more “biologically plausible” by including realistic assumptions. These assumptions are largely derived empirically, but some aspects of good models may be induced from first principles. For example, it is a necessary (but not sufficient) assumption that models should be formulated in a way that is internally consistent. Principles such as symmetry and invariance, which have played a large part in the induction processes of physicists, may eventually become more commonplace within other branches of science.

Currently, the scale of neuroimaging data is too large for a completely coherent Bayesian generative model of inter-subject variability to be adopted. In practice, Bayesian modellers need to make a number of assumptions in order to properly deal with the uncertainty with which parameters may be estimated. Even the simplest of these approximations (the Laplace approximation) is currently too computationally expensive for the scale of model needed for anatomical MRI scans. However, such models are being developed for relatively small datasets (Allassonnière et al., 2007) and the exponential growth in computer power may make them practical within a few more years. Fully Bayesian generative models, such as Deep Belief Nets (Hinton et al., 2006), that currently work well with lots of two dimensional images of order 32 × 32,^2^ may eventually become a reality for MRI data, which contain about 10,000 times as many pixels. Until then though, a reasonable compromise is likely to be from a feed-forward approach, where features are identified using approximate generative modelling strategies (i.e. not fully Bayesian), and these features are fed into a pattern recognition model.

There are several approaches that simply estimate the most probable values of model parameters, which are known as *maximum a posteriori* (MAP) estimates. Although formulated from a generative modelling perspective, they are not truly Bayesian because they do not properly consider the uncertainties of the parameter estimates. Modelling this uncertainty is necessary for making accurate probabilistic predictions, or for making inferences about levels of significance. Currently, the most widely used data analysis strategy within the neuroimaging field involves using a series of models, such that information derived from fitting a lower-level model is fed as input into the model at the next level. These models are colloquially known as “tools”, and each application of a tool is a pre-processing step in an analysis pipeline. The final step in the pipeline (the highest-level model) is the one that answers the question posed by the investigator, which is often formulated within the SPM framework (Friston et al., 1994). In such approaches, information about the question of interest is not fed backwards into the pre-processing steps. For example, when spatially normalising images prior to comparing a number of populations of subjects, the scans would all be treated identically and aligned with the same template — irrespective of their group memberships. In principle, the effects that will later be modelled as confounds in the general linear model could be fed back without biasing the findings, but unless a fully Bayesian approach is adopted, including knowledge about effects of interest would lead to incorrect inferences.

Similar approaches will be described in this review, whereby approximate generative models are fitted to the original data in order to capture useful lower-level features encoding inter-subject variability. This is done without feedback from the top level, so there is no influence from information pertaining to the effects of interest. Features derived from this model are then entered into a completely independent pattern recognition scheme to characterise those aspects of inter-subject variability that are of most interest to the investigator.

The paper is aimed at investigators who wish to model their data, but whose areas of expertise may lie elsewhere. Relatively little mathematical notation is used, but appropriate references are provided for those wishing to read further. Wherever possible, we have attempted to explain the ideas in an intuitive way using graphical illustrations. The next section will describe some of the principles behind multivariate pattern recognition, but with an emphasis on probabilistic approaches. This will be followed by a section about some of the kinds of generative models that may be used for extracting features for use in multivariate modelling of inter-subject variability.

## Multivariate pattern recognition

Many of the analyses of anatomical MRI data are carried out by clinical researchers, where the emphasis is often towards translational or applied research. In many cases, the goal involves characterising the anatomical difference between two populations of subjects. A commonly used approach is to localise volumetric differences of particular brain structures or tissue types, for example by using voxel-based morphometry (VBM) (Wright et al., 1995). Approaches such as this allow the investigator to identify regions of significant difference among the pre-processed data. In the case of VBM, providing the tissue classification and inter-subject alignment models are sufficiently accurate, findings may be interpreted as regional volumetric differences (Ashburner and Friston, 2001).

Other ways of characterising differences exist (Petersson et al., 1999), which do not require the features to be discretely localised. Sometimes such characterisation may be formulated to answer different kinds of questions, such as those about the link between patterns of brain asymmetry and schizophrenia (e.g. Chance et al., 2005), or the extreme male brain theory of autism (Baron-Cohen, 2002). In terms of meeting the aims of a study, the empirical success of a model could be defined in terms of how well it is able to separate the populations. By removing the artificial assumption of independence among brain regions, it is often possible to achieve much greater accuracy. The independence assumption is convenient for localising differences, but empiricism shows that it is not always realistic (Mechelli et al., 2005; Xu et al., 2008; Seeley et al., 2009). Some forms of anatomical variability cannot be localised to specific regions. Consider distinguishing male from female human faces as a typical example for understanding biological variability. This is something that most of us can do intuitively, without being explicitly aware of the pattern that separates them. Buried among all the inter-subject variability that is unrelated to sex, there is a global pattern of difference based on proportions of various measurements, which cannot be localised to particular parts of the face. Similarly, much of the anatomical variability among brains cannot be localised. For example, where would one localise a pattern of difference where the total volume of the left hemisphere is inversely correlated with the volume of the right hemisphere? Volumes of structures are correlated among different brain regions, especially between homotopic regions in the contralateral hemisphere (Mechelli et al., 2005). Patterns of growth are partially predicted by patterns of gene expression, and the gene expression maps at, for example, the Allen Brain Atlas^3^ show spatially distributed patterns. Darwin noted that there is correlation of growth in his *Origin of Species*, so such pleiotropic effects should be expected. Connectivity among brain regions, as well as numerous other factors, is also likely to lead to such spatially distributed correlations. Findings from localisation approaches are relatively simple to explain within the constraints of the journal paper format, but they may only provide approximate summaries of the real pattern of difference.

### Multivariate models

Orthodox linear multivariate techniques such as Principal Component Analysis (PCA), Canonical Correlation Analysis (CCA) and Multivariate Analysis of Variance (MANOVA), have been used by the brain imaging field for a number of years, for modelling both functional (Fletcher et al., 1996; Friston et al., 1996; McIntosh et al., 1996) and structural (Bookstein, 1996; Ashburner et al., 1998) data. Similarly, morphometric applications of multivariate models have existed for many years, and there are several textbooks available on the subject (Small, 1996; Bookstein, 1997; Dryden and Mardia, 1998; Kendall et al., 1999; da Fontoura Costa and Cesar, 2001; Lele and Richtsmeier, 2001; Krim and Yezzi, 2006; Davies et al., 2008). Earlier morphometric approaches involved the application of multivariate statistics to manually defined landmarks or surfaces, often after correcting the data for pose and size using a *Procrustes analysis*. These techniques were considered revolutionary at the time (Adams et al., 2004), but they are relatively naïve when compared with some of the current state-of-the-art computational anatomy models. The re-discovery of Bayesian methods, as well as the additional computer power that has become available, has both contributed to many of the advances. What was once PCA, is now probabilistic PCA (Tipping and Bishop, 1999b; Bishop, 1999). CCA has now been re-formulated as probabilistic CCA (Bach and Jordan, 2005). Many of these models are now treated as components to much larger models, so there are now mixtures of PCAs (Tipping and Bishop, 1999a; Ghahramani and Beal, 2000) and a very wide variety of other models (Roweis and Ghahramani, 1999) that generally fall under the domain of *machine learning*. Pattern recognition is the form of machine learning that will be touched on in this review. Interested readers, requiring more depth, are referred to some of the many good textbooks on the subject (Duda et al., 2001; Bishop et al., 2006).

The basic idea behind pattern recognition approaches is that a number of examples of training data are presented to the model, where each of the examples has some label associated with it. The algorithm then attempts to learn the relationship among data and labels, so that it may predict the desired labels if novel examples of data are presented. A practical application may involve the data being some set of image features for each of a number of subjects, and the labels may be either zero or one, depending on some disease status. In this situation, the model would attempt to automatically make diagnoses based on the image features of the new subjects. Similar approaches have been used for functional data, for which introductory and tutorial papers such as Mitchell et al. (2004) and Pereira et al. (2009) may be helpful.

It is useful to consider each subject's set of features as a single point (a vector) in a high-dimensional space. This is much easier to visualise in situations where there are only two features per subject, as the points can be plotted in two dimensions. Visualisation with three features is also possible, but it becomes extremely difficult if there are four or more dimensions to conceptualise.

**Fig. 1:**
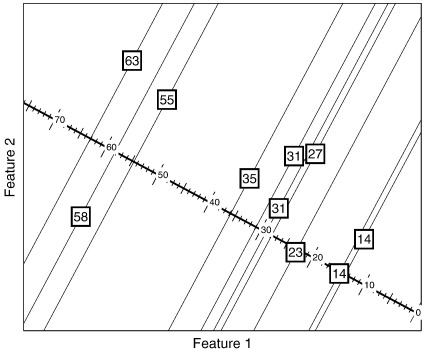
A two dimensional illustration of a regression model, whereby the horizontal and vertical positions of the squares denote the values of pairs of features, and the numbers in the squares indicate labels to be predicted. After fitting the model, prediction is achieved by projecting the data.

**Fig. 2:**
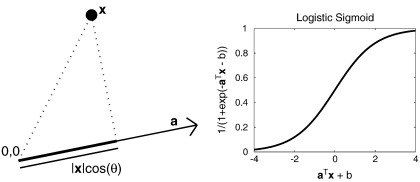
Left: A dot-product can be conceptualised as projecting one vector on to another. Projecting on to the discriminating direction is by **a**^*T*^**x** = |**a**||**x**| cos(*θ*), where *θ* is the angle between **a** and **x**. Right: A logistic function is used for squashing the results into the range of zero to one (because they are probabilities).

In addition to classification into discrete categories, pattern recognition may be used to predict continuous variables. This is known as regression, and Fig. 1 provides a simple illustration. After fitting the model to the training data, predictions for new data may be made by *y* = **a**^*T*^**x** + *b*, where **a**^*T*^**x** is a dot-product operation, illustrated in Fig. 2. Rather than simply predict the most probable value of *y*, more advanced regression methods would predict a distribution, which may be encoded by the mean and standard deviation of a Gaussian.

**Fig. 3:**
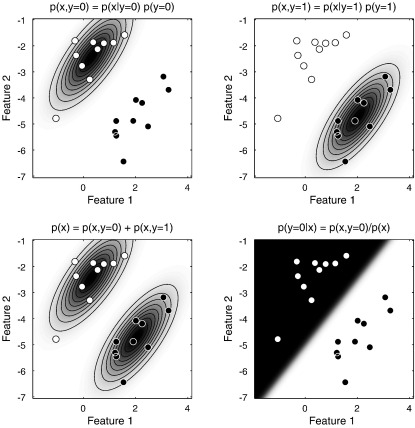
A two dimensional illustration of the generative model used by Fisher's linear discriminant analysis.

**Fig. 4:**
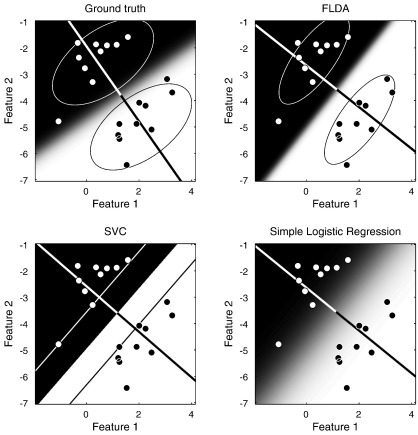
This figure shows a selection of some of the approaches that can be used for linear discrimination. Top-left: Ground truth is based on the probability densities of the two Gaussians from which data were simulated. The line shows the discriminant direction for the underlying model. Top-right: Fisher's Linear Discrimination, also including the resulting discriminant direction. Bottom-left: Linear Support Vector Classification results. Bottom-right: A simple logistic ridge-regression model.

**Fig. 5:**
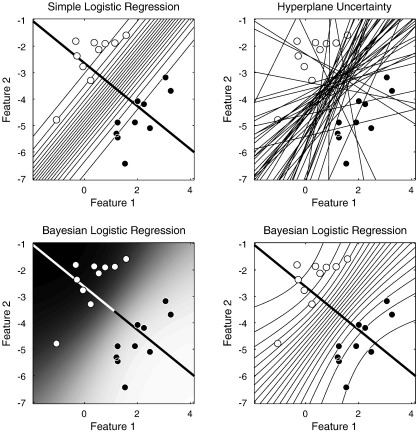
This figure illustrates a Bayesian approach to logistic regression. Top-left: Contours of probability from a naïve implementation of logistic regression, where the contours remain parallel (see Fig. 4). Top-right: The discriminating direction is estimated with uncertainty, which is illustrated by a random sample of possible separating hyperplanes. Accurate inference requires this uncertainty to be integrated into the predictive model. Bottom-left: Predictive probabilities are made more accurate by incorporating uncertainty. Bottom-right: By integrating out the uncertainty, the contours properly reflect the loss of accuracy further away from the training data.

Many principles will be illustrated for the case of classification, and Figs. 3, 4 and 5 will show schematics for a simple two dimensional example. White circles are intended to denote subjects in group 0, and black circles denote those in group 1. The two axes represent the values of the two features, where the features could be measurements such as the volumes of particular structures. Data for a new subject could then be plotted, whose group membership is unknown. If a subject's data is closer to the white circles, then it may be more likely to belong to group 0. If it is closer to the black ones, then group 1 may be more likely. In the case of a simple comparison between two groups of subjects, the objective would be to partition the space into two regions. New data falling into the first region would be assigned membership to the first group, whereas if it falls into the second region then it would be assigned to the second group. In practice, these assignments may not be made unambiguously, so the partitioning would be probabilistic.

### Generative and discriminative models

Fisher's Linear Discriminant Analysis (FLDA) is a commonly used, but simple, framework for multivariate modelling of data. FLDA is a special case of Canonical Correlation Analysis (Bach and Jordan, 2005), but it may also be viewed as a special case of a Mixture of Gaussians (MoG), which in turn is a special case of other more complicated models. Essentially, FLDA involves a model whereby there are two populations of data, both sharing the same multivariate Gaussian variance. FLDA can be considered a *generative model*, as it attempts to encode a probability density of the entire dataset. Referring to the illustration in Fig 3, a generative model would encode the probability density of the two classes of data. In the case of FLDA, this involves representing them as multivariate Gaussian distributions, shown in the two sub-figures at the top. The probability of belonging to a particular class is then obtained by dividing by the probability density of the data itself, which is simply the sum of the probabilities of belonging to the various classes (two in this example). This is a simple application of Bayes rule:

$$p\left(y=0|x\right)=\frac{p\left(y=0,x\right)}{p\left(x\right)}=\frac{p\left(x|y=0\right)p\left(y=0\right)}{p\left(x|y=0\right)p\left(y=0\right)+p\left(x|y=1\right)p\left(y=1\right)}\text{.}$$

Modelling the difference between one group of data and the other requires the within group variance and covariance to be modelled. For this reason, it is often necessary to use some form of dimensionality reduction. Images may consist of millions of voxels, whereas the number of subjects in a study is usually much fewer than this. For FLDA to work effectively, the millions of voxels would need to be reduced to fewer features than there are subjects. This problem is known as the *curse of dimensionality*, and there are a number of techniques that may be used for factorising a large dataset into its most salient components. PCA is a commonly used approach for this, but there are a number of more principled alternative models that could be chosen. For such studies, the within group variability is not of primary concern, and all that is required is an accurate characterisation of the salient differences among the groups.

Rather than use a generative model, it is possible to directly estimate the separation between the groups using a *discriminative model*. Within population variance is not usually considered interesting, and modelling it requires additional parameters that are difficult to deal with optimally. An analogy to using a generative model for classification would be needing to learn both Mandarin and German in order to distinguish between spoken versions of the two languages. It is simpler to directly identify the distinguishing features. The approaches are also known as the *diagnostic paradigm* (discriminative) and the *sampling paradigm* (generative) (Hand, 2001). In most practical situations, discriminative models are more accurate and robust than generative models (Bishop et al., 2006) (except for small training datasets), so they should provide more accurate characterisations of the differences among populations of subjects.

Generative models for discrimination do offer some advantages over discriminative models (Lasserre et al., 2006). In particular, for probabilistic approaches it is much more straightforward to make use of additional unlabelled data within a generative modelling framework. Zhu and Goldberg (2009) and Chapelle et al. (2006) provide useful references for such semi-supervised learning strategies. A related situation occurs when group memberships of training data are not known with 100% confidence, where it may be helpful to use probabilistic labels for training the model. For example, definitive diagnosis of Alzheimer's disease (AD) is only possible from post-mortem samples. Training a system to identify AD may be more optimal if the labels are able to encode the fact that (for example) some subjects have an 80% probability of having the disease. By considering FLDA within a MoG framework, it should become possible to assign probabilistic labels to the training data in much the same way as tissue probability maps are currently used to provide priors for tissue classification (Ashburner and Friston, 1997; Van Leemput et al., 1999). Discriminative models may also be able to make use of such probabilistic labels, but the authors are not yet aware of any related work. It is often easier to formulate domain knowledge about a system using generative modelling strategies, and there is now an increasing degree of interest in combining generative and discriminative training (Lasserre et al., 2006; Bishop and Lasserre, 2007; Schmah et al., 2008), such that the best attributes of both may be exploited.

Domain knowledge is often incorporated by pre-processing, which is a form of generative modelling. It is then possible to use the model to compute similarity measures among the pre-processed observations, using concepts from *Information Geometry* (Amari and Nagaoka, 2007), such as *Fisher kernels* (Jaakkola et al., 2000; Holub et al., 2008). The relationship between geometry and shape analysis is clear, but it may not be so apparent that the Information Geometry framework also extends to other generative models used for pre-processing. Fully Bayesian generative models, which combine pre-processing and classification into the same probabilistic model, could offer advantages in terms of feeding information back to lower levels of the model (Mumford, 1994, 2002; Hinton et al., 2006).

### Probabilistic approaches

For classification, the objective is sometimes simply to divide the space of possible data into binary regions, such that new data is categorised as belonging to one group, or the other. There are various approaches to do this, which include the support-vector machine (SVM) (Boser et al., 1992; Cristianini and Shawe-Taylor, 2000; Schölkopf and Smola, 2002) that is applied increasingly to anatomical neuroimaging data (Golland et al., 2001; Golland, 2002; Davatzikos et al., 2004; Lao et al., 2004; Golland et al., 2005; Fan et al., 2005; Kloppel et al., 2008b; Vemuri et al., 2008; Davatzikos et al., 2008; Magnin et al., 2009; Gerardin et al., 2009). SVMs are based on Vapnik's *Statistical Learning Theory* (Vapnik, 1999), and often perform very well in binary classification problems. The principles behind SVMs have been described in many neuroimaging papers, so no further details will be provided here. Other approaches, such as relevance vector classifiers (RVCs) (Tipping, 2001) and other logistic regression techniques, attempt to provide probabilistic predictions. Examples of such approaches, applied to the same data as in Fig. 3, are illustrated in Fig. 4. For linear methods, such classifications are performed by *y* = *f*(**a**^*T*^**x** + *b*), where *f*() is some non-linear function. The function would be a simple thresholding procedure for SVM classification, whereas it would be a logistic function for logistic regression models (Fig. 2). Whether a new datum is assigned to one class or the other, will depend on which side of a separating hyperplane it falls. In two dimensions, such a hyperplane is one dimensional, whereas in three dimensions, it is two dimensional. It always has one less dimension than the dimensionality of the data, and is essentially defined by the vector orthogonal to it (**a**), and a scalar that indicates where it intersects the vector (*b*). Refinements can be made to the simple logistic regression model, as well as to RVCs, in order to make their predicted probabilities more accurate. Without the refinements, although they attempt to make probabilistic predictions, these models tend to be over-confident for novel data that is far from any encountered during training (Rasmussen and Quinonero-Candela, 2005). Fig. 5 attempts to show that by integrating out the uncertainty in the estimation of the discriminative direction, it is possible to counteract this over-confidence.

These illustrations were only for two dimensional data, and there were many more data points than dimensions in the data. Linear regression involved fitting a general linear model, where the aim is to determine the optimal linear combination of data that best predict the labels. For logistic regression, estimating the separating hyperplane could be done by using a general*ised* linear model (GLZ) to fit the data to the labels. This is similar to fitting a general linear model (GLM), except that it involves the use of a link function to squash the output within the range of zero and one (see Fig. 2). Finding the solution requires an iterative approach, which is typically an iterative re-weighted least squares. It is also worth noting that the logistic regression model can be generalised to discriminate among multiple classes, by fitting a Softmax function (Bishop et al., 2006).

### Gaussian process models

**Fig. 6:**
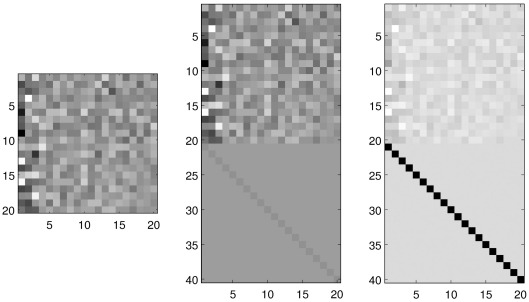
A poorly-determined general linear model may have a number of columns in the design matrix that is high, compared to the number of rows (left). The conditioning of the problem may be improved by augmenting the design matrix (centre and right). In this situation, fitting the GLM involves a trade-off between fitting the data and keeping the coefficients small. If the regularisation part is small (centre), the trade-off is towards the former, whereas if it is large (right), then model will tend towards the latter.

The usual approach for modelling neuroimaging data involves fitting a linear combination of columns in a design matrix (independent data) to fit a single vector of image data (dependent data). For pattern recognition, the model is reversed because training involves modelling the independent data by a linear combination of the dependent data (see e.g. Friston et al. (2008) for further explanations). Unlike the case for non-linear models (Friston and Ashburner, 2004; Hoyer et al., 2009), it makes no difference to most linear models whether the independent and dependent data are swapped around, providing any confounding effects are properly modelled. When making predictions about subjects from their image data, it is not possible to use a simple GLM (for regression) or GLZ (for classification), as each image has far more voxels than there are images in the dataset. This is the curse of dimensionality, which was touched on earlier, and requires some form of regularisation in order to resolve the fact that the model is under-determined. As mentioned previously, one form of regularisation involves reducing the data to a smaller number of salient features. A more elegant strategy involves penalising the coefficients in the GLM using a ridge-regression technique, which essentially adds additional prior knowledge into the system of equations. The objective then becomes one of optimising the fit to the label data, while simultaneously keeping the sum of squares of the coefficients as small as possible. This involves a trade-off between bias and variance in the model's ability to generalise to new data, which is controlled by the hyper-parameters of the model (see Fig. 6). With too little regularisation, the model will fit the training data very well, but the predictions for new data may not be accurate because it has over-fitted the training data. In contrast, with too much regularisation, the model will be strongly biased towards classifying everything with 50% probability (or whatever the proportions of group members are in the training data). Achieving an optimal solution involves determining the optimal balance between fitting the training data and penalising the magnitudes of the coefficients. The older literature suggested a number of ad hoc methods for this, but the Bayesian framework provides a more elegant solution in the form of the evidence framework (MacKay, 1992) (which is the same as type-II maximum likelihood, empirical Bayes and restricted maximum likelihood). By integrating out the uncertainty with which the coefficients (parameters) are estimated, the evidence framework essentially estimates only hyper-parameters.

For regression, estimating the hyper-parameters is equivalent to maximising the probability of the *N* training labels (**y**) under the assumption that they are drawn from a zero mean Gaussian distribution, where the covariance matrix (**C**) is computed as some function of the training data. The covariance matrix is parameterised by the hyper-parameters, which are determined by maximising the probability according to the equation for a multivariate Gaussian distribution:

$$p\left(y|C\right)=\frac{1}{\sqrt{{\left(2\pi \right)}^{N}|\det C|}}\exp\left(-\frac{1}{2}y^{T}C^{-1}y\right)\text{.}$$

There are many ways of parameterising the covariance matrix, but the main criterion is that it needs to be symmetric and positive semi-definite. A simple model with three hyper-parameters is:

$$C=\theta_{0}I+\theta_{1}+\theta_{2}XX^{T}\text{.}$$

In this case, *θ*_0_ would add some amount of a diagonal matrix of ones to the covariance matrix, which models residual variance. A constant offset in the regression is accounted for by the *θ*_1_ term, which models the variance of the *b* in *y* = **a**^*T*^**x** + *b*. The *θ*_2_ term encodes the variance of the regression coefficients (**a**). If there are *N* subjects in the training data, then the matrix **XX**^*T*^ is an *N* × *N* matrix, which encodes the similarities among the scans. Each set of features may be treated as a row vector, and these *N* rows would be stacked together to form the matrix **X**. The matrix **XX**^*T*^ contains the dot-product of each of the *N* feature sets, with each other feature set in the training data. Training involves finding the values for *θ*_0_, *θ*_1_ and *θ*_2_ that maximise the probability of **y**. After training, it is then possible to use those estimated values to build a larger covariance matrix, encompassing both the training feature sets as well as the features to test. The augmented covariance matrix, in conjunction with the training labels, can then be used to predict the unknown labels. This is the Gaussian process regression framework (Williams and Rasmussen, 1996), and is nicely described in textbooks such as MacKay (2003),^4^ Rasmussen and Williams (2006)^5^ or Bishop et al. (2006). There is also a related framework for classification (Williams and Barber, 1998), although practical implementation is not so straightforward, and often involves a number of approximations.^6^ The important point here, is that Gaussian process classification also requires a covariance matrix, which is formulated in the same way as that for regression.

#### Feature selection

If some features of the data are known to be less informative than others, then it is possible to down-weight their importance. Similarly, if it is known, a priori, that a sparse set of features are likely to provide the most accurate predictions, then the pattern recognition algorithm may be modified such that it is more likely to select a sparse set of features. A number of authors have devised feature selection procedures for applying pattern recognition to imaging data. The objective of feature selection is to ignore, or down-weight, those features that provide relatively less discriminatory signal. Within the Gaussian process framework, this would be analogous to ignoring the contribution made, by those features, to the matrix of dot-products.

This kind of naïve feature selection may be formulated using a diagonal matrix, **W**, which is a function of several, non-negative, hyper-parameters (*θ*_2_, *θ*_3_, etc). The limiting case of this framework would be the situation where each element on the diagonal of **W** was one of the hyper-parameters. This is known as *automatic relevance determination*, and usually results in sparse solutions as some of the hyper-parameters fall to zero. Solutions obtained from this model are equivalent to those described in Friston et al. (2008). For this kind of model, the covariance matrix would be given by:

(1) $$C=\theta_{0}I+\theta_{1}+XW\left(\theta_{2},\theta_{3}\ldots \right)X^{T}\text{.}$$

Sparsity may be over space, but this need not be the case. It is possible that pre-processing models, such as independent component analysis (Bell and Sejnowski, 1995), could transform the data into the kind of features whereby selecting a sparse subset would add biological plausibility to the discrimination. Similarly, there are other factorisation models that could prove useful for defining the kinds of features where sparsity would be advantageous. One such example may be non-negative matrix factorisation (Lee and Seung, 1999).

There is no reason why **W** should be limited to the diagonal case. For example, if the features consist of image data that have been pre-processed in some way, then **W** could encode a convolution function, such that the algorithm may determine the optimal degree of spatial blurring. It is often the case that low spatial frequencies contain proportionally more informative signal than do the higher frequencies, so more accurate predictions may be obtained by blurring the data by some optimal amount.

Later, the paper will explain a possible framework for this type of approach, whereby a similar **W** matrix may be used to obtain a trade-off between information pertaining to shape, and information pertaining to image intensity.

#### Going non-linear

Sometimes, it is not possible to achieve accurate predictions using a linear separation method, in which case non-linear methods may be required. For example, a particular disorder may be characterised by a number of alternative types of variability. A simple example would be a disorder that either causes atrophy in the left or in the right hemisphere. A linear model would only be able to encode one mode of variability, whereas a non-linear model may be able to capture both modes.

Non-linear models work by projecting the data into a higher number of dimensions, where they can be fitted using a linear model (see e.g., Cristianini and Shawe-Taylor (2000), Bishop et al. (2006)). This is similar to the use of polynomial expansions for simple non-linear fitting of data. There is a class of methods, known as *kernel methods*, that is ideally suited to this approach. These methods use the *kernel trick*, which involves replacing the matrix of dot-products (**XX**^*T*^) by some other symmetric and positive semi-definite matrix, which is a function of the data. One of the most widely used forms for this matrix is one based on radial basis functions (RBF), which requires distances between all pairs of feature vectors. It is possible to derive distances from matrices of dot-products because (*x*_1_ − *x*_2_)^2^ = *x*_1_^2^ + *x*_2_^2^ − 2*x*_1_*x*_2_. Each element of the matrix would then be replaced by $\exp\left(-\frac{\theta }{2}d_{\mathrm{mn}}^{2}\right)$, where *d* is the distance between feature set *m* and feature set *n*, and *θ* is a hyper-parameter controlling the width of the kernel.

Rather than use simple Euclidean distances between each pair of feature sets, it is also possible to use other measures of distance within the RBF framework. The only requirement is that the measures must satisfy the requirements for being a *metric*, which are:

1. They must be greater than or equal to zero.
2. They may only be equal to zero if the features are identical.
3. The distance between **x**_*m*_ and **x**_*n*_ must be equal to the distance between **x**_*n*_ and **x**_*m*_.
4. The distance between **x**_*m*_ and **x**_*n*_ must not be greater than the sum of those between **x**_*m*_ and **x**_*k*_, and between **x**_*k*_ and **x**_*n*_.

A strategy for deriving metrics between shapes will be described later. Many pattern recognition procedures can be formulated as kernel methods, but several other algorithms can also be kernelised.

Non-linear methods allow more complicated separations to be achieved, but they also make it easier for the model to over-fit the training data. As in the case of the previous examples, the hyper-parameter(s) controlling the degree of non-linearity may be automatically determined using the evidence framework. Also, interpreting the mechanism by which separation is achieved is much more difficult when non-linear methods are used (Golland, 2002). Ideally, linear methods would be used whenever possible, but this may require representing the features in a form that allows easier separation using a linear method.

Real data often falls in manifold-like patterns within the high-dimensional space (see e.g. Baloch and Davatzikos (2009)), but the careful use of generative models may allow much of this pattern to be modelled. A simple example would be an image of a brain that is rigidly transformed by various amounts. There are six parameters controlling the rigid transform, so the transformed versions of the image would fall as points on a six-dimensional manifold. Rigid-body alignment could be used to bring all the points on the manifold back to a single point. Similarly, inter-subject registration methods are able to model out some of the manifold-like patterns within MRI data. Unlike the rigid-body alignment case, the way that the spatial transformation is encoded is also of interest because it describes the shape of the brain. To be as powerful as possible, pattern recognition for studies of inter-subject variability should be formulated to work both with shape descriptors, and also with the variability that cannot be modelled out by alignment.

### Measuring empirical success

Many classification methods are able to fully separate the training data, whereas they may generalise poorly to novel data. Often, cross-validation is used to assess how accurately the characterisation separates the populations.^7^ This may involve fitting the model to all subjects' data except for one, and then assessing the accuracy of the prediction about the subject that was left out. The procedure would be repeated by leaving out the next subject's data, and so on. Reports of sensitivity and specificity, using measures such as the area under the receiver operating characteristic (ROC) curve (see e.g. Zou et al. (2007)), can be compared with those of human experts. Human expertise is still considered to be the gold standard to beat for most models encoding image understanding (Kloppel et al., 2008a). This situation may change over the next few years, as computing power will probably continue to grow exponentially (“Moore's Law”), thus allowing new levels of algorithmic sophistication.

In other situations, the comparisons tend to be among computer models, and cross-validation is less likely to be used. Measures, such as the Bayesian model evidence, minimum description length (MDL), Bayesian information criterion (BIC) and the Akaike information criterion (AIC), may be used for assessing how well models encode probability densities, thus allowing the evidence for different models to be compared, so that the best model for each particular dataset may be selected. Real Bayesian modellers do not use cross-validation, and the neuroimaging field is seeing the increasing use of evidence measures for choosing among models (Penny et al., 2004; Beckmann and Smith, 2004; Friston et al., 2007; Behrens et al., 2007; Friston et al., 2008; Kiebel et al., 2008). Providing all the model assumptions are met, Bayesian strategies for model selection are more efficient – both in terms of computational complexity, and in terms of the available degrees of freedom (Rasmussen and Williams, 2006) – than cross-validation for selecting the optimal model from a number of candidates.

Cross-validation is often used for optimising feature selection, or for adjusting other settings in the model. If there are not too many settings to adjust, then a grid search strategy can be used, where a range of settings are tried, and the most successful is chosen. However, within the Bayesian modelling framework, this type of approach can be greatly streamlined. The best model is the one with the highest model evidence, and the model fitting procedures are geared towards searching over the space of possible hyper-parameters in order to maximise this measure.

### Large datasets

Some investigators object to the Bayesian view of modelling because it involves the use of prior knowledge. Other approaches, such as orthodox statistical techniques, also involve some form of subjectivity — but this subjectivity is usually hidden. For example, what is so special about a value of 0.05 when assessing the significance of a *p* value? Should a correction for multiple comparisons be used when interpreting findings from multiple studies? For Bayesian methods, the relative effect of the priors becomes less important for large datasets, so findings become less subjective as more data are modelled.

Bayesian model selection strategies try to identify the most appropriate model for the data, and the optimal choice relates to the quantity and quality of data. As more becomes available, the complexity of the optimal model will continue to increase until it reaches that of the system under study. In the case of biological systems, this complexity is likely to exceed that of typical datasets. For this reason (and others), the sharing and re-utilisation of valuable and well characterised data is likely to become increasingly important for the integrative models that are required for research in both systems biology and for translational work (Van Horn and Toga, 2009). Relatively few investigators build on primary data from previous fMRI studies because of the subjectivity of the stimuli used, and the difficulties inherent in attempting to organise the experiments into any useful structure. In contrast, as demonstrated by ADNI (Mueller et al., 2005; Butcher, 2007) and other similar projects (Marcus et al., 2007), the primary data required for studies of anatomical variability tend to be re-used and built on extensively. Just as data generated by the Human Genome Project would be relatively worthless if only one investigator had access to it, the same may be true of primary data from large studies of neuroanatomical variability. If data is of sufficiently high quality and relevant, then others will wish to use it. Measures such as the *h*-index are becoming increasingly important as measures of productivity (rather than simply the numbers of publications) (Hirsch, 2007). Not only would data-sharing increase transparency and reproducibility of the scientific process, but it is also a way to help maximise the impact of work. Many funding bodies now require some sharing of primary data, and terms such as “mega-analysis” are beginning to enter the vocabularies of neuroimagers (Salimi-Khorshidi et al., 2009). Inevitably, some researchers will object to mixing data from different scanners, sequences etc, claiming that models of data collected on one scanner cannot be generalised to data from another. This is not an argument against pooling data, but is instead a quite different one.

Given the exponentially increasing ease with which genes can be sequenced (and the exponentially decreasing cost), sharing primary data is likely to become especially important for future studies attempting to link genotype with phenotype. A search through hundreds of thousands, or even millions, of single nucleotide polymorphisms (or – within a few years – entire genomes) presents a colossal multiple comparisons problem. For this type of work, the aim would be to find those alleles that have the greatest measurable effect (any effect) on brain anatomy (or function). Identifying those genetic associations that best predict neuroanatomical variability will require multivariate modelling of very large datasets. Another approach to finding further clues about the causes of various disorders would be to generate a multivariate characterisation of the typical pattern of deviation from a control population. This pattern may be expressed to various degrees in the healthy population, which leads to the possibility of probing for the pattern in a large population of genetically characterised subjects.^8^

## Generative modelling

Data are usually pre-processed by modelling them generatively in order to derive useful features, which are subsequently fed into the discriminative framework. This section deals with certain aspects of *Pattern Theory*, which is a generative modelling framework that begins with the premise that real world patterns are complex, and that encoding this complexity should be allowed (Mumford, 1996; Mumford, 2002; Grenander and Miller, 2007). Simplifying organisational principles may emerge from such complex models, but these would not be discovered unless the data is modelled in all its complexity. Currently, much of Pattern Theory concerns shape modelling, although it is an area of research that is likely to expand and subsume a wider variety of probabilistic models. In principle, Pattern Theory is not really so different from other reputable Bayesian modelling strategies, but its ambitions may be greater in that it was formulated to deal with the kind of complexity encountered in biological systems. The ideas proposed within this review differ from the Pattern Theory perspective in that the generative modelling is treated as a pre-curser to a discriminative modelling step. Pattern Theory would involve a single unifying generative model for everything.

Models based on Pattern Theory are also formulated to accommodate various symmetries and invariances. In this respect, the models are closer to those used by theoretical physicists, but allowing for a much greater amount of complexity. A framework for deriving metrics from *diffeomorphic* mappings will be introduced later. This framework is a component of Pattern Theory, and is based on a kind of *exponential mapping* procedure. First though, some simple illustrations of ordinary exponentiation will be presented.

### Allometry

Linear regression or classification based on original data is not always possible. Sometimes, some form of modelling can be used to transform it so that linear (or less non-linear) methods can be successfully applied. This section introduces the use of logarithms as a very simple pre-processing procedure, which, in turn, is a prelude to a later section of the paper.

**Fig. 7:**
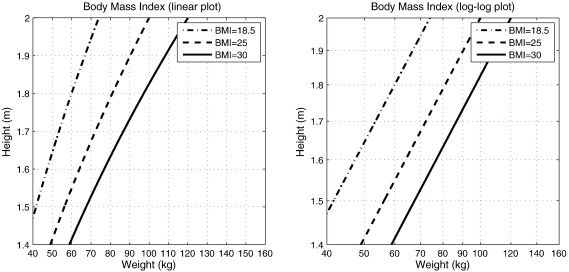
An illustration of allometric relationships using BMI as an example.

A widely used and convenient marker for obesity is Quetelet's Body Mass Index (BMI), which is defined as the weight of the subject (in kg) divided by the square of their height (in m). Fig. 7 shows contours for different BMIs, which appear curved on a plot of height against weight, but straight when the logarithm of height is plotted against the logarithm of weight. We may also note that weights are not scaled with the cube of height (i.e. the relationship is not isometric), so ideal body proportions differ in a systematic and predictable way according to size. The relationship among the data can be expressed in a way that would be more intuitive for prediction by a multivariate model.

logBMI=logweight−2logheight.

The field of studying the relationship among logarithms of measures is known as *allometry*. Unconstrained growth can be considered a process of self-replication (Huxley, 1993), and the logarithms of volumes, lengths etc can be seen as revealing more about the “causes” of the measurements, than the measures do themselves. The final shape of an organ can be modelled as the result of some pattern of differential growth rates, and the logarithms tell us something about these rates. When relating the magnitude of one measurement (*y*) to another (*x*), it is common to express the relationship by *y* = *bx*^*k*^, where *k* and *b* are constants. An alternative way of expressing the relationship is by log *y* = log *b* + *k* log *x*. The parameter *b* is of little biological significance, whereas *k* (the exponent) can be considered as a measure of the relative growth.

A number of investigators have related brain weight with body weight among different species. For example, (Martin, 1981) noted that from a sample of 309 species of placental mammals, log_10_ *y* = 1.77 + 0.76 log_10_ *x*, where *y* is brain weight and *x* is body weight. For a sample of 11 species of anthropoid primates, the relationship between brain volume and body weight was found to be log_10_ *y* = 1.36 + 0.71 log_10_ *x* (Rilling, 2006). Rilling (2006) also noted that the exponent of allometry relating cortical surface area and brain volume of primate brains is around 0.8, which is greater than the value of 0.67 that would be expected if brains varied isometrically. This work relates to that of (Zhang and Sejnowski, 2000), who devised an allometric model for grey and white matter volumes in mammalian brains. Larger brains contain proportionally more white matter, which has been confirmed using MR scans of human brains by (Luders et al., 2002). Sometimes, simple patterns can emerge from complex systems. One of the essential assumptions in allometric scaling theory is that convergent evolution leads to nearly optimal systems with similar gross characteristics (West et al., 2001; Csete and Doyle, 2002; West and Brown, 2005).

In situations where no prior data are available, the Bayesian framework allows the use of uninformative priors. Where variables are real values that may be negative as well as positive, an uninformative prior would assume that all values, both positive and negative, are equally possible.^9^ Where variables may only be positive, a different strategy is used for assigning priors. Here, the probability of a value being between one and 10 is equal to the probability of it being between 10 and 100, or 0.001 and 0.01. This type of prior is uniformly flat for the logarithm of the variable. It should be noted that the products of positive real values are also positive real values, and that the sum of the logarithms of positive real values is also the logarithm of a positive real value. In other words, they form what, in mathematics, is called a *group*. Group theory provides a principled mechanism by which to assign priors (Jaynes and Bretthorst, 2003), and is one of the cornerstones of Pattern Theory.

The use of logarithms to transform the measurements may allow the discovery of interesting relationships among data, via the application of linear pattern recognition. Where there are multiple measurements, the multivariate relationship could be expressed by log *y* = log *b* + ∑ _*j*_ _=_ _1_^*J*^ *w*_*j*_ log *x*_*j*_. Unfortunately, there are certain problems in applying a simple allometric model to shape measures, which occur because growth is not unconstrained, and neighbouring or overlapping structures need to grow together. Huxley (1993) pointed out that the logarithm of the volume/mass of a structure should be related to the volume/mass of the whole organism minus that of the structure. Similar concerns were identified by (Zhang and Sejnowski, 2000), who related grey matter volume to white matter volume, and also grey matter volume to the sum of grey and white matter volume. If the relationship that log *y* = log *b* + *k* log *x* holds, then it is not possible for log *y* = log *b*′ + *k*′log(*x* + *y*) also to hold. Resolving this inconsistency requires a different model to account for such correlations. That model may be the one based on the group of diffeomorphisms.

### Identical functions of very different coordinate systems

Merriam-Webster's Medical Dictionary defines *morphometry* as the quantitative measurement of the form especially of living systems or their parts, where *form* means the shape and structure of something as distinguished from its material. The study of form is largely derived from the generative model of D'Arcy Thompson (Thompson and Bonner, 1942), who stated that ‘diverse and dissimilar fish [brains] can be referred as a whole to identical functions of very different coordinate systems, this fact will of itself constitute a proof that a comprehensive “law of growth” has pervaded the whole structure in its integrity, and that some more or less simple and recognizable system of forces has been at work’.

Conventionally, the neuroimaging field treats inter-subject variability as different functions of near-identical coordinate systems. fMRI studies, involving comparisons among populations of subjects, usually attribute their findings to what may be referred to as “functional variability”, whereas many of the results could equally be attributable to variability of the underlying anatomy. Interpretations of exactly what is meant by functional variability may include variability of the magnitude of activations, or activations occurring within non-homologous structures. Unfortunately, the very definition of what constitutes a homology is unclear, which makes it difficult to draw any sharp distinction between “functional” and “anatomical” variability.

Literal adherence to Thompson's model would have implications for how functional data should be used to further our understanding of inter-subject variability. Such a model would require that fMRI be used as a way of labeling the various regions of functional specialization, thereby allowing image registration procedures to bring these labeled regions into alignment (Saxe et al., 2006; Sabuncu et al., 2009). Studies of inter-subject variability could then be based upon the relative shapes of the brains, as estimated by registration algorithms.

Similarly, diffusion weighted MRI could provide information that allows more accurate measurement of relative shape (Behrens et al., 2003, 2006; Klein et al., 2007; Johansen-Berg et al., 2005). Under an assumption that brains all have the same pattern of major tracts, it would appear reasonable to align the brains based on their tracts and simply compare the resulting shapes. A number of approaches are already being developed to align brains using diffusion data (Alexander et al., 1999; Guimond et al., 2002; Ruiz-Alzola, 2002; Park et al., 2003; Zhang et al., 2006). It is common for investigators to want to compare the positions of tracts among spatially normalised images, but findings from such an analysis would essentially be about mis-registration. This is useful for evaluating image registration models, but would not necessarily be considered interesting from a physiological perspective.

In reality, the pure D'Arcy Thompson model may over-exaggerate the importance of form to our understanding of variability. Language lateralization provides a clear example of where such a model would fail, as it involves patterns of functional specialization that could clearly not be modelled by shape differences alone. Because the term “homologous” is only vaguely defined, future advances to our understanding of variability may be more likely to arise from models that have the potential to combine form-like and function-like variance, in an elegant and parsimonious manner.

Shape models are an important component of the feature sets used for pattern recognition (Golland et al., 2001, 2005), but features based on residual differences after registration are also of potential importance (Makrogiannis et al., 2007), particularly if information from fMRI or diffusion imaging is to be included within the model. It is possible that increased power may be achieved by using a more sophisticated model for these patterns of residual variability (Trouvé and Younes, 2005). There is much that could be said on the subject of template models of the brain and how they relate to this pattern, but it would be beyond the scope of this review. In the next sections, we will try to explain how the residual differences after registration can actually be used to encode deformations (Younes, 2007). The registration model that is needed for achieving this goal appears rather more complicated than most, but it may have the potential to simplify the feature sets used for multivariate analysis.

### Diffeomorphic shape models

**Fig. 8:**
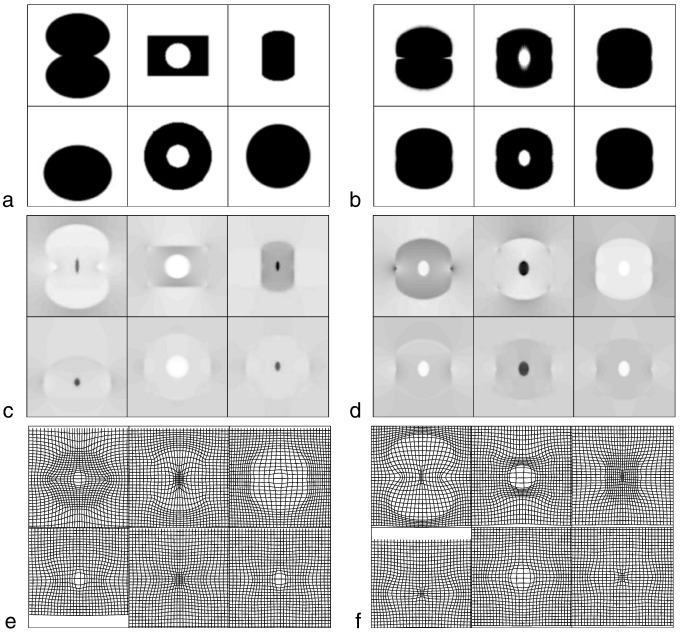
The original images used for this illustration are shown in (a). After alignment with their common average, they are shown in (b). Note that exact alignment is not achieved, especially for the white hole in the middle of two of the images. Decreasing the amount of regularisation used by the registration would have allowed the hole to be closed further, but its area would never reach exactly zero (a singularity). The Jacobian determinants indicate the relative volumes before and after non-linear registration. Lighter colours indicate areas of expansion, where the Jacobians are smaller. Darker colours indicate contraction, and larger Jacobians. A Jacobian determinant of one would indicate no volume change. The Jacobians of the mapping from the original images to the warped versions are shown in (c). The diffeomorphic framework allows deformations to be invertible, so mappings from the warped images to the originals can also be generated. The Jacobians of these mappings are shown in (d). The deformations and their inverses are shown in (e) and (f). Spatially normalised versions of the individual images were generated by resampling them according to (f), whereas (e) could be used to overlay the template on to the original images. The forward and inverse mappings can be composed together, in which case the results should be identity transforms (which would appear as a regular grid).

A *diffeomorphism* is a smooth, one-to-one mapping, and the diffeomorphic framework developed by Miller, Younes, Grenander and others (Miller et al., 1997; Grenander and Miller, 1998; Miller, 2004; Grenander and Miller, 2007; Miller and Qiu, 2009) is potentially very useful for modelling shapes of brains. There is a large literature on mathematical shape models, but much of it is aimed at mathematicians, and may not be accessible to investigators who do not have a solid mathematical background. In this section, we try to provide a more intuitive understanding of some of the principles that underlie these developments, attempting to convey their importance with as little mathematical notation as possible. To further simplify the explanations, the principles will be illustrated using the simple two dimensional example images shown in Fig. 8. These images were aligned to their common average shape, where this involved iteratively alternating between recomputing a template and re-estimating the warps that map between the template and the original data. With this simple model, computing the template image involved generating a pixel-wise weighted average of the warped images, where the weighting is by the Jacobian determinants of the warps. These Jacobians indicate the amount of local expansion or contraction incurred by the non-linear deformations. After registration, the volume of a structure in each of the original images can be estimated by summing the Jacobians over the region of template containing the structure. A key feature of diffeomorphic registration methods is that the Jacobians cannot become negative, which ensures that estimated volumes are also never negative.

In theory, diffeomorphic deformations have a number of useful properties. When two diffeomorphic deformations are composed together, then the result is diffeomorphic. If multiple diffeomorphisms are composed, then it does not matter whether it is done as *A* ∘ (*B* ∘ *C*) or (*A* ∘ *B*) ∘ *C*. A diffeomorphic mapping that is conceptually useful sometimes, is the identity transform. When this is used to warp an image, then the image remains the same. With diffeomorphic registration, there should be no folds in the deformations and all Jacobian determinants should be positive. Folding would indicate that the one-to-one mappings have broken down (Christensen et al., 1995). Because they encode one-to-one mappings, diffeomorphisms also have inverses. All these properties mean that diffeomorphisms form a mathematical group.

**Fig. 9:**
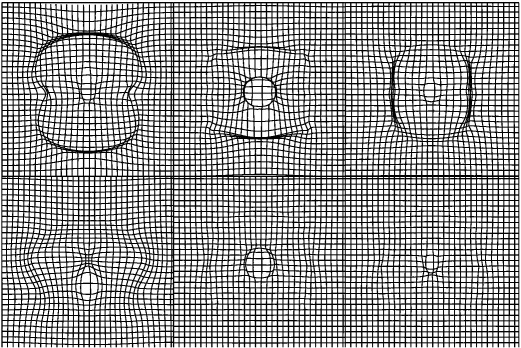
The small deformation framework is not accurate for larger deformations. This figure shows the sum of the forward and backward displacement fields shown in Fig. 8. The results are clearly not identity transforms.

For a pair of numbers close to one, it is possible to approximate multiplying them together by subtracting one from each of them, adding the results together and adding back one. For example, the result of 1.02 × 0.995 × 1.003 can be approximated by 0.02 − 0.005 + 0.003 + 1 (to give 1.018, instead of 1.0179447). This approximation becomes less accurate as the numbers deviate further away from one. The small deformation framework, which most investigators use for working with deformations, is similar to this approximation. It involves subtracting an identity transform, working with some linear model of the resulting displacement fields and then adding the identity transform back again. This approximation may be reasonable when displacements are very small, but is less accurate when the deformations are larger — a point illustrated in Fig. 9. Shapes are the ultimate non-linear sort of thing (Mumford, 2002), and building accurate models requires some more sophisticated mathematics.

Allometry involves treating an original measurement of length, area, volume etc as the exponential of a growth rate. If a structure begins with a volume of one, and grows at a constant rate of *k*, then its final value after one unit of time will be exp(*k*). Similarly, the framework for diffeomorphisms involves treating the deformation of objects as a kind of exponential mapping (*Riemannian exponential mapping* — see e.g. Younes et al., 2009). In this case, the deformation begins as an identity transform (no deformation), and the object deforms at a constant rate over unit time. The procedure considers the evolving deformation as a dynamical system, and the rate of deformation can be considered analogous to the logarithms in the allometric framework.

#### Distance measures for non-linear pattern recognition

One technique for comparing shapes in a non-linear multivariate way is to use metrics (Trouvé and Yu, 2001). These are measures of distance between points, which satisfy a number of requirements. Often when we consider distances, we are dealing with linear spaces, but there are many instances when the underlying space is curved (non-linear). A simple example would be a distance between two world cities, where the shortest path between them, tangential to the surface of the globe, would serve as a metric (see earlier). The earth's surface can be thought of as a two dimensional manifold, embedded within a three dimensional Euclidean space. The paths would follow *geodesics*, which are defined as the (locally) shortest distances between points in a curved space. When working with scans of different subjects, the idea would be to have a measure of distance between each pair of scans. Such metrics lend themselves easily to the use of radial basis function kernel pattern recognition algorithms, as the elements of the kernel matrix are simply a function of distance between pairs of images. Alternatively, there are ways of classifying a new point based on finding the closest points in the training data, and making the assignment based on which group they belong to (K-nearest neighbour).

Pattern recognition and other multivariate methods have been applied to metrics derived by inter-subject registration (Miller et al., 2008). These metrics may be thought of as measures of the distance travelled by one brain as it is warped to the shape of another, but they are not based on simple lengths of trajectories of the voxels. Instead, the measures consider the relationships among the trajectories of neighbouring voxels. For example, if a set of neighbouring voxels move in parallel with each other, the distance is likely to be shorter than if they move in different directions. If their motion remains parallel, then this is simply a uniform displacement — rather than a shape change. If they move in different directions, then this results in a change of shape. Defining distances this way provides a measure of smoothness of the deformations, and hence the amount of distortion. There are many ways to specify metrics between anatomies, and the choice may depend on the application (Mumford, 2005). Shapes can vary in different ways, and the accuracy of pattern recognition algorithms can be helped by knowing what kind of measures are most likely to be informative. This is the *Ugly Duckling Theorem*^10^ (Watanabe, 1969), which says that all objects are equally similar to each other, unless the importance of certain distinguishing features is known a priori.

Image registration algorithms such as *Large Deformation Diffeomorphic Metric Mapping* (LDDMM) (Beg et al., 2005) may be used for measuring these distances between shapes. LDDMM is a volumetric image registration procedure, which aligns images by minimising the sum of squares difference between them, while keeping a metric distance as short as possible. Although LDDMM was formulated in a continuous way, it can be conceptualised as an algorithm that estimates a series of small deformations, which are composed together to give a large diffeomorphic deformation. The objective is to estimate the entire series of deformations, such that the total measure of “energy” in the small deformations is as small as possible. Elegant mathematics underlying the formulation of LDDMM mean that the locally shortest distance (*geodesic distance*) may be found by minimising this total energy. These distances may serve as metrics, which may be used for non-linear pattern recognition.

With current technology, such an approach is too computationally expensive for routine use, as it requires each pair of images to be registered together. For example, if there are 100 subjects in a study, then 5050 registrations are needed to obtain the metric between each pair of subjects' data. For this reason, this review will focus on a slightly different framework, which is based on approximating the curved space by assuming that it is locally flat (i.e. working on the *tangent space*).

#### Local linear approximation methods

Approximating a curved space by a linear (flat) space involves introducing distortions, which need to be overcome as far as possible. For example, projections of the globe on to a flat two dimensional map (e.g. by the Mercator projection) incur distortions, but the centre of the map tends to be less distorted than those regions towards the edges. Reducing the amount of distortion in a linear approximation can involve centering the origin of the flat coordinate system at a suitable location. When considering shapes as points on some manifold, the least distortion would be achieved by centering the origin at the “average” of those shapes (Woods, 2003). It would seem intuitive that warping individual subjects to an average shaped template would reduce bias in an analysis, rather more than warping all subjects to match a randomly selected individual from the group (Beg and Khan, 2006). However, an average in a non-linear space is more difficult to compute than an average in a Euclidean (flat) space, and can be achieved by minimising the sum of squares of the geodesic distances (metrics) between the mean, and each of the individual points. This approach is implicitly used by a number of group-wise volumetric registration models (e.g. Joshi et al., 2004; Avants and Gee, 2004; Lorenzen et al., 2005).

**Fig. 10:**
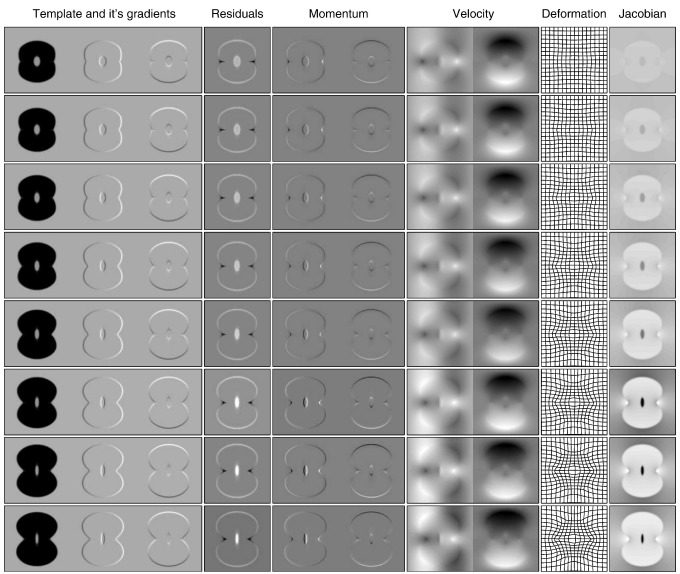
Deformations can be generated from the residuals as illustrated here (Younes, 2007). The top row shows the initial state of the system, and each subsequent row shows it at the next time point during the evolution. The bottom row shows the final state. The first three columns show the template and its spatial gradients as it evolves to match the individual image. The next column shows the residual difference between the template and warped image, scaled to account for contraction and expansion.

Once all images have been aligned with their average shaped template, interesting features may then be derived from them. Diffeomorphic mappings have a number of properties that makes them potentially useful for analysis of inter-subject variability. The LDDMM algorithm estimates a mapping between images by minimising a geodesic distance between them, but estimating the same mapping can be formulated from a different perspective. Using a procedure is known as *geodesic shooting*, it is possible to derive the entire mapping from the initial velocity with which the template would be deformed at a constant rate over unit time. The mathematics are too deep to enter into details here, but Fig. 10 attempts to illustrate the evolution of the dynamical system for one of the images in Fig. 8. The underlying mathematics are explained, from a number of perspectives, by Miller et al. (2006), Cotter and Holm (2006), Marsland and McLachlan (2007), Younes (2007), and Younes et al. (2008, 2009). Referring to Fig. 10, it is possible to conceptualise the evolving deformation as the composition of a series of very small deformations. The last two columns of the figure show the evolving deformation, and the relative amount of expansion or contraction incurred at each point. The displacements of each small deformation are given by the velocity fields shown in the seventh and eighth columns of the figure. The velocity is a vector field, with horizontal and vertical components, and is obtained from the “momentum” by convolving it with a suitably smooth function (Bro-Nielsen and Cotin, 1996).

**Fig. 11:**
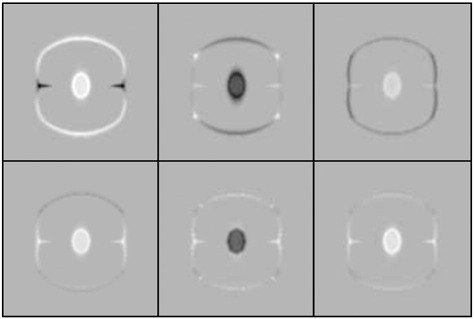
This figure shows residual differences between the warped images and the template, which are scaled at each point by the Jacobian determinant. In conjunction with the template, these residuals encode the information needed to reconstruct the original images (apart from a small amount of information lost through inexact interpolation). Dividing the residuals by the Jacobian determinants and adding the template will give warped versions of the originals, which can then be unwarped by resampling with the appropriate deformation. The deformations and Jacobians needed to perform these operations are actually encoded by the residuals (illustrated in Fig. 10).

The main point to be made here, is that the momentum may be computed from a map of residual differences multiplied (voxel by voxel) by the spatial gradients of the template. The same template is warped into alignment with each of the images, so its contribution to the initial velocity is the same for all of the individual images. The thing that differs among individuals is the map of residuals. Given the template, the individual images are fully specified (give or take some interpolation error) by their associated residuals. Fig. 11 shows these residuals for all the individual images. It should be noted that much of the information in these maps of residuals is in alignment across images. This and the fact that they are scalar rather than a vector fields, along with their sparsity over space, would suggest that they offer a very parsimonious way of encoding the variability of the deformations (Younes, 2007). Not only do these maps encode deformations, but they also encode the residual differences after alignment.

At this point, it is important to stress that the simple residual images do not offer the most optimal shape-based features. In other words, simple dot-products among pairs of residual images would not generate a kernel matrix that would lead to the most accurate pattern recognition. A measure of similarity between the shapes of two objects would be computed from the dot-product of the initial velocity for one shape, and the initial momentum of the other. Momentum may be derived via a matrix multiplication of the residuals by a matrix **G** (where the matrix consists of diagonal matrices containing the gradients of the template). Similarly, the convolution operation for computing initial velocity from initial momentum may also be conceptualised as a multiplication with matrix **K**. For further details of this approach, see e.g. Vaillant et al. (2004), Wang et al., 2007, and Qiu and Miller (2008). Returning to the Gaussian process modelling framework, the similarity among shapes may be expressed as **XWX**^*T*^, where **X** encodes the residual differences and **W** subsumes all the matrices for generating the necessary dot-products of momentum and velocity: **W** = **GKG**^*T*^. In principle, the above framework could be extended further such that other aspects of the residuals could also contribute. A simple example would be **W**(*θ*_2_, *θ*_3_) = *θ*_2_**GKG**^*T*^ + *θ*_3_**I**, where **I** is an identity matrix. Many variations on this same theme could be developed.

A similar framework is also applicable for other objective functions used as matching criteria (Younes et al., 2009). For example, fMRI or diffusion MR data could be included within the registration procedure, which would allow the inter-subject variability of both form and function to be combined within the same model. Previously, (Makrogiannis et al., 2007; Baloch and Davatzikos, 2009) used a pattern recognition framework, whereby both maps of residual differences between warped individuals and the template, as well as deformation fields were used as features. A balance was sought between the contributions made by the residual differences between aligned tissue class images and template, and the deformation fields. This section has tried to show that the residual differences themselves, after scaling by the Jacobian determinants of the deformations, are enough to encode the deviation of the original images from the template.

**Fig. 12:**
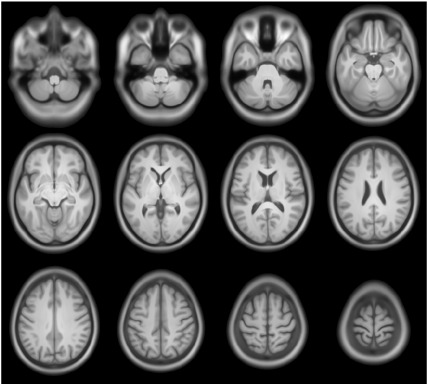
Average of 450 T1-weighted scans from the IXI dataset, which have been aligned using a geodesic shooting model. The left side of the brain is shown towards the left of the image.

Brain images can be registered volumetrically using this way of encoding relative shape, and an example of an average is shown in Fig. 12. The entire dataset^11^ from the EPSRC funded IXI dataset were diffeomorphically aligned, except for subjects IXI012 and IXI050. Registration used a similar procedure to that described by (Ashburner and Friston, 2009), except that the opimised parameters encoded initial velocities for geodesic shooting, rather than the constant velocity parameterisation used by Dartel (Ashburner, 2007). When averaging, the images were intensity normalised so that their average intensities were identical, and the Jacobian determinants were used to weight the average. Note that regions outside the brain appear blurred as registration was only based on simultaneous alignment of grey and white matter.

### Visualising differences

One of the major problems of multivariate techniques is in interpreting the pattern of findings. Unlike mass-univariate approaches, these are global and not localised to discrete regions. For various reasons, the neuroimaging field may be reluctant to accept such a framework, but multivariate morphometric approaches are widely accepted within other biological domains for making comparisons among species. A model of anatomical variability that works well between species should also be applicable within species.

Although it has not yet been clearly demonstrated for anatomical brain images, it is likely that models that are multivariate over space may be more accurate than those that model each voxel independently. The main challenge to be overcome for multivariate morphometric studies concerns visualising and communicating the findings, which is probably why so many geometric morphometric studies of the brain have focussed on simple two dimensional structures such as the corpus collosum. Three dimensional volumes are quite difficult to visualise, especially within the limited space of most journals. This is further complicated by the fact that patterns of difference are often vector or tensor fields, which are quite difficult to visualise in two dimensions, but in three dimensions the problem becomes much worse. In comparison, differences localised by voxelwise methods can be easily presented in the form of statistical parametric maps, particularly if relatively few differences are identified. Although the reasons may appear trivial, voxelwise models will probably continue to dominate because their results can be explained and presented much more easily.

For linear classifiers, it is possible to represent the discriminative direction (Golland, 2002; Golland et al., 2005) by a vector that has the same dimensionality as the data features of each individual. This would be the vector **a** in the expression *y* = *f*(**a**^*T*^**x** + *b*). Non-linear classification presents an additional problem, in that any attempt at encoding a discriminative direction will only be an approximation (Golland, 2002). The reason for this is that the separating hyperplane is curved, so the direction perpendicular to it will vary from place to place.

Representations of shape differences from surface-based models may often be visualised more easily, especially if relatively simple structures are modelled. For example, (Golland et al., 2001) shows displacements by first projecting them in the directional perpendicular to the surface (see Fig. 2). In fact, with the appropriate diffeomorphic registration model, any momentum differences will be perpendicular.

**Fig. 13:**
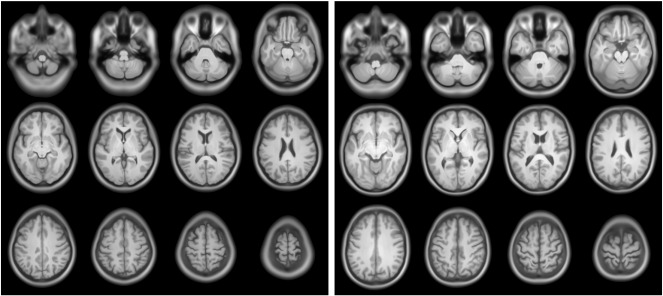
Exaggerated versions of female (left) and male (right) average brains, which correspond to 99.99999% probabilities. Note that the caricatures were generated by warping the average brain shown in Fig. 12, and that the deformations outside the brain are less accurate (so skull thicknesses etc are not accurately represented). The left side of the brain is shown towards the left of the image.

So far, relatively little work has been carried out on how best to visualise and communicate multivariate patterns of difference. One possible approach may be to generate caricatures. Fig. 13 shows exaggerated versions of male and female brains from the IXI dataset (Fig. 12), which were generated using a geodesic shooting method. Pattern recognition was by fitting a regularised logistic regression model using a linear kernel matrix, based on dot-products of initial momentum and initial velocity (Vaillant et al., 2004; Wang et al., 2007). Scans in the IXI dataset were collected on three different scanners. Scanner and subject ages were included as a confounds within the model (so three subjects with unknown ages were excluded), so the optimisation involved parameterising a covariance matrix by

$$C=\theta_{0}I+\theta_{1}+\theta_{2}XGKG^{T}X^{T}+\theta_{3}SS^{T}+\theta_{4}aa^{T}$$

where **X** encodes the residuals, **S** encodes which scanner was used and **a** encodes the ages of the subjects. Multiplication with matrix **K** is essentially the same as convolving with the same smooth function used by the image registration algorithm. The discriminating direction was identified from the model, in terms of an initial velocity. From this, it was possible to determine how far to deform the average shaped brains along the discriminating direction, such that the shape was either male or female, with 99.99999% probability. A geodesic shooting method was used to evolve the shapes, which meant that the exaggerated deformations did not lose their one-to-one mapping.

## Conclusion

This paper has emphasised a Pattern Theoretic perspective on modelling neuroimaging data, with some compromises in terms of treating the parts of the model for “pre-processing” separately from those parts used for making statistical inference. Fully Bayesian generative models of inter-subject variability are not yet practically feasible. Instead, a generative model is used for pre-processing, and a discriminative model is used for making predictions.

It usually requires several decades for basic science to become applied science, so it is worth considering which basic science approaches have the greatest potential for translation. Over the shorter term, translation is likely to require accurate models of inter-subject variability in order to fully utilise the information that is available within MR scans. Some of the more immediate applications are for diagnostics and biomarkers, and for localising abnormalities. Other applications may involve registering useful atlas-based information on to scans of individuals for the purpose of pre-surgical planning. All these examples require accurate models of inter-subject variability, as well as a useful framework in which to formulate a variety of questions.

Computer power is currently doubling approximately every year and a half, which implies a ten-fold increase in speed every five years. If this trend continues, processing speeds will increase by a factor of one hundred in ten years, ten thousand in twenty years, and by a million in thirty years. So far, only a few tentative steps have been made towards applying machine learning techniques to neuroscience and medicine. Given enough processing power and good scientists working together to develop increasingly accurate models, there is no reason why computers could not play a much larger role in future.

## Footnotes

1 This was the main conclusion of the New York Academy of Sciences “What Do We Want to See in Brain Imaging?” meeting (London, UK. 3–4 December, 2007).

2 See http://yann.lecun.com/exdb/mnist/ for the accuracy with which handwritten digits can be recognized using various pattern recognition approaches.

3 http://humancortex.alleninstitute.org/.

4 This book is freely available at http://www.inference.phy.cam.ac.uk/mackay/itila/.

5 This book is freely available at http://www.gaussianprocess.org/gpml/chapters/.

6 See http://www.gaussianprocess.org/ for some implementations, and other useful information.

7 Cross-validation is exactly analogous to the hypothesis testing approach that is commonly accepted within the field, except that the hypotheses have been learned by a pattern recognition algorithm.

8 Geoffrey Tan, personal communication.

9 Technically, these priors are “improper”. Probability densities must integrate to one, so a probability density allowing an infinite range of possible values must have zero probability everywhere.

10 It is called the “Ugly Duckling Theorem” because a swan and a duckling are just as similar to each other as two swans.

11 http://www.ixi.org.uk.
